# Automated Detection of Acute Myocardial Infarction Using Asynchronous Electrocardiogram Signals—Preview of Implementing Artificial Intelligence With Multichannel Electrocardiographs Obtained From Smartwatches: Retrospective Study

**DOI:** 10.2196/31129

**Published:** 2021-09-10

**Authors:** Changho Han, Youngjae Song, Hong-Seok Lim, Yunwon Tae, Jong-Hwan Jang, Byeong Tak Lee, Yeha Lee, Woong Bae, Dukyong Yoon

**Affiliations:** 1 Department of Biomedical Systems Informatics Yonsei University College of Medicine Yongin Republic of Korea; 2 VUNO Inc Seoul Republic of Korea; 3 Department of Cardiology Ajou University School of Medicine Suwon Republic of Korea; 4 Center for Digital Health Yongin Severance Hospital Yonsei University Health System Yongin Republic of Korea

**Keywords:** wearables, smartwatches, asynchronous electrocardiogram, artificial intelligence, deep learning, automatic diagnosis, myocardial infarction, timely diagnosis, machine learning, digital health, cardiac health, cardiology

## Abstract

**Background:**

When using a smartwatch to obtain electrocardiogram (ECG) signals from multiple leads, the device has to be placed on different parts of the body sequentially. The ECG signals measured from different leads are asynchronous. Artificial intelligence (AI) models for asynchronous ECG signals have barely been explored.

**Objective:**

We aimed to develop an AI model for detecting acute myocardial infarction using asynchronous ECGs and compare its performance with that of the automatic ECG interpretations provided by a commercial ECG analysis software. We sought to evaluate the feasibility of implementing multiple lead–based AI-enabled ECG algorithms on smartwatches. Moreover, we aimed to determine the optimal number of leads for sufficient diagnostic power.

**Methods:**

We extracted ECGs recorded within 24 hours from each visit to the emergency room of Ajou University Medical Center between June 1994 and January 2018 from patients aged 20 years or older. The ECGs were labeled on the basis of whether a diagnostic code corresponding to acute myocardial infarction was entered. We derived asynchronous ECG lead sets from standard 12-lead ECG reports and simulated a situation similar to the sequential recording of ECG leads via smartwatches. We constructed an AI model based on residual networks and self-attention mechanisms by randomly masking each lead channel during the training phase and then testing the model using various targeting lead sets with the remaining lead channels masked.

**Results:**

The performance of lead sets with 3 or more leads compared favorably with that of the automatic ECG interpretations provided by a commercial ECG analysis software, with 8.1%-13.9% gain in sensitivity when the specificity was matched. Our results indicate that multiple lead-based AI-enabled ECG algorithms can be implemented on smartwatches. Model performance generally increased as the number of leads increased (12-lead sets: area under the receiver operating characteristic curve [AUROC] 0.880; 4-lead sets: AUROC 0.858, SD 0.008; 3-lead sets: AUROC 0.845, SD 0.011; 2-lead sets: AUROC 0.813, SD 0.018; single-lead sets: AUROC 0.768, SD 0.001). Considering the short amount of time needed to measure additional leads, measuring at least 3 leads—ideally more than 4 leads—is necessary for minimizing the risk of failing to detect acute myocardial infarction occurring in a certain spatial location or direction.

**Conclusions:**

By developing an AI model for detecting acute myocardial infarction with asynchronous ECG lead sets, we demonstrated the feasibility of multiple lead-based AI-enabled ECG algorithms on smartwatches for automated diagnosis of cardiac disorders. We also demonstrated the necessity of measuring at least 3 leads for accurate detection. Our results can be used as reference for the development of other AI models using sequentially measured asynchronous ECG leads via smartwatches for detecting various cardiac disorders.

## Introduction

Wearable devices, simply referred to as “wearables,” are smart electronics or computers that are integrated into clothing and other accessories that can be worn on or attached to the body [1]. The consumer adoption of wearable technology for health care services is skyrocketing owing to increasing interest in personalized health management, disease prevention, and fitness [2,3]. One such technology is continuous/day-to-day measurement of single-lead electrocardiograms (ECGs) via smartwatches or other portable/handheld devices [4-6]. These devices can provide a novel opportunity for facilitating timely diagnostics by extending the availability of ECG measurement to the general population outside the hospital.

Smartwatches and other portable/handheld ECG devices measure single-lead ECG when the 2 electrode detectors are attached to 2 different parts of the body [5]. However, useful information from other leads can potentially be neglected when only a single lead is evaluated [7]. Analyzing electrical activity of the heart from different spatial locations by measuring multiple leads is necessary for accurate and robust detection of cardiac disorders, such as myocardial infarction, pulmonary embolism, and acute left or right heart failure [8,9]. Accordingly, the standard 12-lead ECG is the most commonly used assessment among physicians for evaluation of the heart.

**Figure 1:**
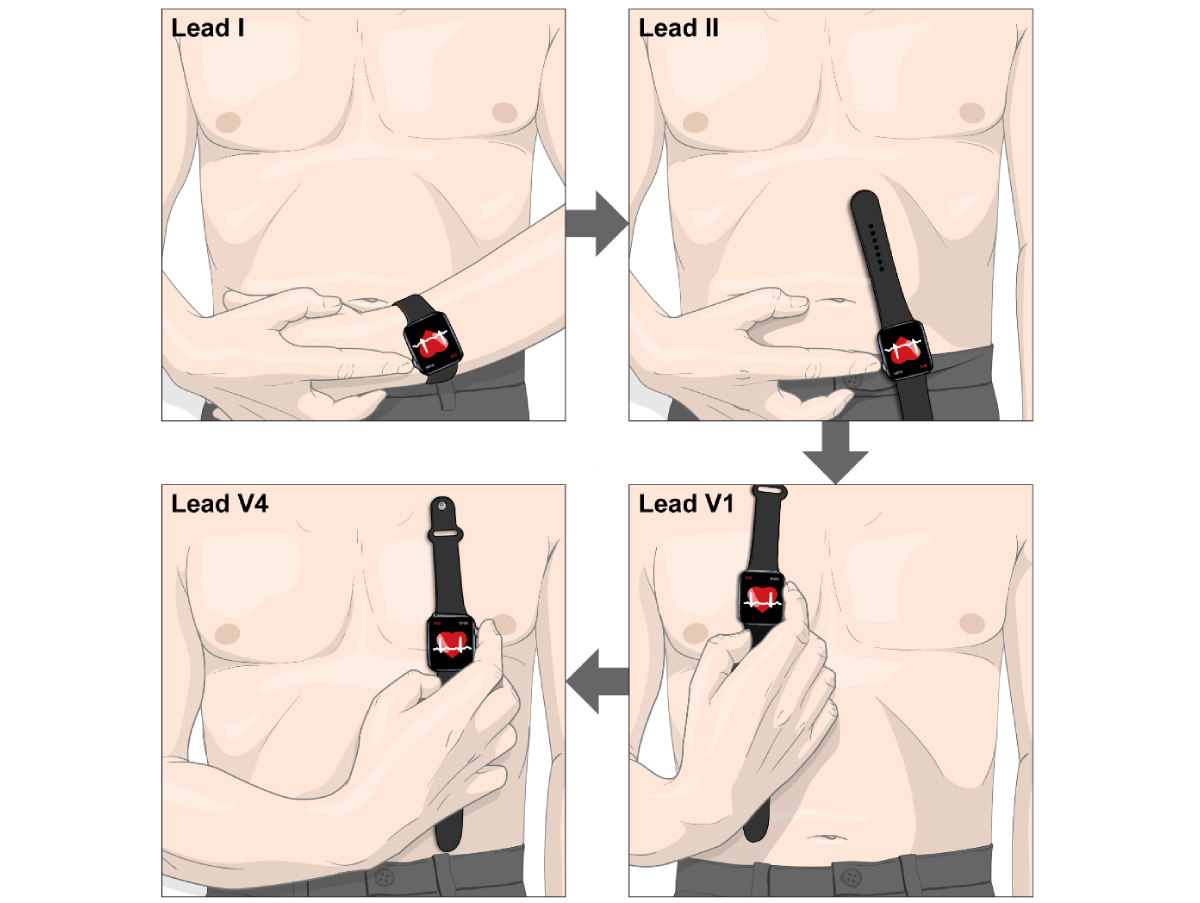
Example of measuring multi-lead electrocardiogram (ECG) from a smartwatch. Multiple-lead ECG can be obtained from smartwatches by sequentially placing the smartwatch on different parts of the body. The figure depicts an example of measuring leads I, II, V1, and V4 sequentially. Lead I can be recorded with the smartwatch on the left wrist and the right index finger on the crown. Then, after removing the smartwatch from the left wrist, lead II can be recorded with the smartwatch on the left lower quadrant of the abdomen and the right index finger on the crown. Next, leads V1 and V4 can be recorded with the smartwatch on the fourth intercostal space at the right sternal border and fifth intercostal space at the midclavicular line, respectively, with the right index finger on the crown in both cases.

Previous studies have explored the possibility and described the methodology of measuring multiple ECG leads using smartwatches [9,10]. Multiple ECG leads can be obtained from smartwatches by sequentially placing the smartwatch on different parts of the body (Figure 1). The ECG signals from different leads are asynchronous when measured in this way. There are also reports evaluating the concordance of multiple-lead ECG obtained by smartwatches compared with the standard 12-lead ECG in detecting conditions related to ischemic heart disease when read by physicians [11-13].

To the best of our knowledge, previous studies on automated diagnosis or classification of ECGs using artificial intelligence (AI) have utilized either single-lead ECGs or synchronous multiple-lead ECG signals as input [14-19]. Application of asynchronous ECG signals for AI model development is largely unexplored. Such an application needs to be assessed to ensure that multiple lead-based AI-enabled ECG models can be implemented on smartwatches. Moreover, the adequate number of sequentially recorded leads from smartwatches that would ensure sufficient diagnostic power of the AI-enabled ECG model needs to be verified.

In this study, we aimed to develop an AI model for detecting acute myocardial infarction using asynchronous ECG lead sets and then compare the performance of our model with that of an automatic ECG interpretation provided by a commercial ECG analysis software. Such a model could prove the feasibility of AI-enabled ECG algorithms on smartwatches. As a prerequisite to develop such a model, we derived asynchronous ECG signals from standard 12-lead ECG reports to simulate a situation similar to the sequential recording of ECG leads via smartwatches. Moreover, we aimed to find the optimal number of leads for sufficient diagnostic power by randomly masking each lead channel during the training phase and validating/testing our model with various targeting lead sets (and masking the remaining lead channels).

## Methods

### Ethics Approval

The Institutional Review Board of Ajou University Hospital approved this study (protocol AJIRB-MED-MDB-20-597) and waived the requirement for informed consent because only anonymized data were used retrospectively.

### Data Sample and Labeling

We utilized standard 12-lead ECG reports collected from General Electric (GE) ECG machines at Ajou University Medical Center (AUMC), a tertiary teaching hospital in South Korea. These ECG reports of AUMC originally exist as PDFs and are stored in a database. Thus far, the ECG database contains a total of 1,039,550 ECGs from 447,445 patients, collected between June 1994 and January 2018. A previous study extracted raw waveforms, demographic information, and ECG measurement parameters/automatic ECG interpretations made by the GE Marquette 12SL ECG Analysis Program from these reports [20]. In these reports, each lead is 2.5 seconds in duration and sampled at 500 Hz. We also collected clinical data, such as emergency room visit time or the diagnosis of the patients, from the AUMC Electronic Medical Records database.

For our study, we identified and extracted ECGs recorded within 24 hours from each visit to the emergency room between June 1994 and January 2018 from patients aged 20 years or older. For each visit to the emergency room, all diagnoses made during the stay in hospital were collected. If either International Classification of Diseases, Tenth Revision (ICD-10) code I21 (acute myocardial infarction) or I22 (subsequent ST elevation and non-ST elevation) was entered, the ECGs for those visits were labeled as having acute myocardial infarction. For visits that had neither of the 2 ICD-10 codes entered, the ECGs for those visits were labeled as not having acute myocardial infarction.

We split the data into training/validation (80%) and independent hold-out test (20%) sets, and then further split the training/validation set into training (85%) and validation (15%) sets. To reduce ambiguity, we excluded patients whose time of registration for the ICD-10 codes for acute myocardial infarction (I21 or I22) was either “null” (meaning that the registration time was not entered and thus is unknown) or not within 24 hours of ECG measurement.

After model development, we compared the performance of our model with that of the automatic ECG interpretation provided by the GE ECG analysis program. To derive the performance of the automatic ECG interpretation for detecting acute myocardial infarction, we categorized the interpretations in 2 different ways. First, the automatic ECG interpretation was categorized as myocardial infarction if the interpretation included at least one of the following three phrases: “ACUTE MI,” “ST elevation,” and “infarct.” The second categorizing criterion consisted of the 3 phrases in the first labeling criterion along with the following three phrases: “T wave abnormality,” “ST abnormality,” and “ST depression.” We thus derived 2 distinct performance indices from these 2 categories.

### Deriving Asynchronous Lead Sets From ECG Reports

Multimedia Appendix 1 shows an example of a standard 12-lead ECG report used at AUMC. These ECG reports are asynchronous as a whole while being synchronous when grouped into 4 subsets of 3 leads each. The x-axis of the ECG report represents time flow; the waveforms on the left side are recorded earlier than those on the right side. The total recorded time of this ECG report is 10 seconds. In Multimedia Appendix 1, leads I, II, and III are shown to have been recorded 2.5 seconds earlier than leads aVR, aVL, and aVF, which were recorded 2.5 seconds earlier than leads V1, V2, and V3, which in turn had been recorded 2.5 seconds earlier than leads V4, V5, and V6.

As previously mentioned, asynchronous ECG lead sets can be derived from ECG reports to simulate a situation similar to the sequential recording of ECG leads via smartwatches. For example, a 4-lead subset consisting of leads I, aVR, V1, and V4 from the ECG report is completely asynchronous. According to the Einthoven law and Goldberger equation, for the 6 limb leads (leads I, II, III, aVR, aVL, and aVF), the remaining 4 leads can be calculated even if only 2 leads are available [21,22]. Here, we trained/validated our AI model by randomly masking each lead channel and then tested our model with various target lead sets (while masking the remaining lead channels) to determine the optimal number of leads for sufficient diagnostic power. The tested lead sets are specified in Multimedia Appendix 2. For the multiple-lead sets, we included lead I in all cases, given that lead I is the most basic lead channel that can be measured from a smartwatch: lead I can be measured by placing the right index finger on the crown without removing the smartwatch from the left wrist. The lead channels in each 4-, 3-, and 2-lead set are completely asynchronous. Thus, the lead channels included in the 4-lead sets were leads I and II (calculated from leads aVR, aVL, and aVF) for the limb leads and all the possible combinations of 2 precordial leads that could be derived from the ECG report while maintaining complete asynchrony. The lead channels included in the 3-lead sets were leads I and lead II (calculated from leads aVR, aVL, and aVF) for the limb leads and 1 precordial lead. The lead channels included in the 2-lead sets were lead I and either lead II (calculated from leads aVR, aVL, and aVF) or 1 precordial lead. We also tested 2 single-lead cases (lead I or II).

### Primary and Secondary Aims of the Study

Our primary aim was to develop an AI model for detecting acute myocardial infarction from asynchronous ECG signals, which outperforms the automatic ECG interpretation provided by the GE ECG analysis program. Our secondary aim was to determine the optimal number of leads required for sufficient diagnostic power. Model performances were assessed using the following statistics: area under the receiver operating characteristic curve (AUROC), area under the precision-recall curve (AUPRC), sensitivity, specificity, positive predictive value (PPV), and negative predictive value (NPV).

### Neural Network Architecture and Training

**Figure 2:**
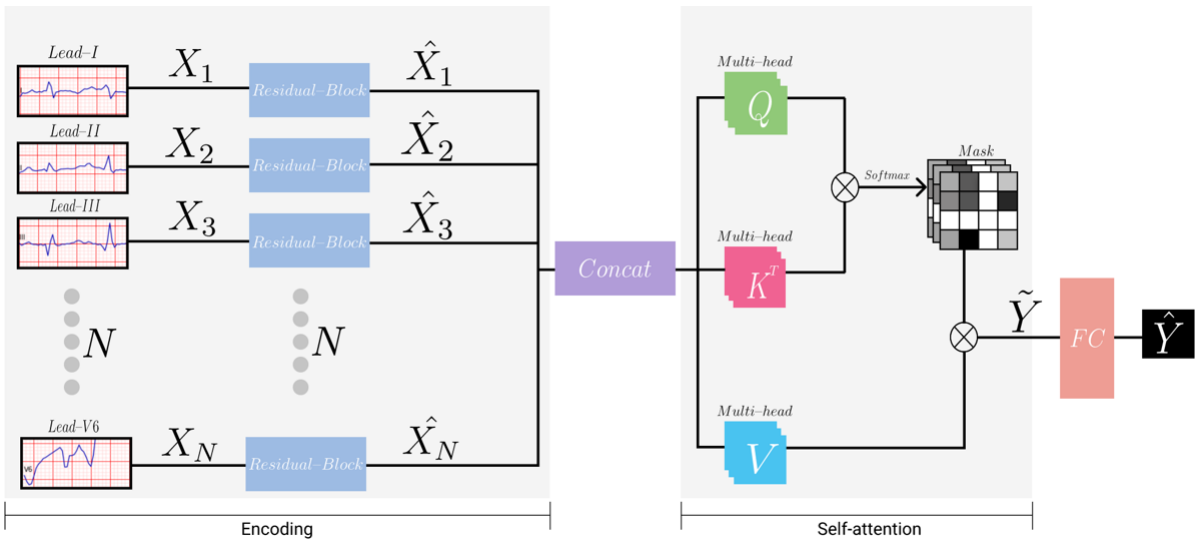
Illustration of the neural network's architecture. The encoding phase encodes each lead channel with a weight-shared structure. The self-attention phase captures the relation between each lead channel.

Figure 2 illustrates the architecture of the neural network model used in our study. The model was divided into two phases: encoding and self-attention.

#### Encoding Phase

The model took the input of 2.5 seconds from each 12-lead ECG channel, which was downsampled from 500 Hz to 250 Hz. Each lead was processed in a separate but weight-shared encoder. Details of the architecture of the encoder are summarized in Multimedia Appendix 3. The encoder consisted of 16 residual blocks with 2 or three 1-dimensional convolutional neural network (CNN) layers in each block [23]. Every CNN layer was followed by a batch normalization layer and a ReLU activation function. All the CNN layers had a kernel size of 7 and the “same” padding. In the first residual block, the output of the first ReLU activation function was connected to the block’s output via a pooling layer. In the following 15 residual blocks, the input and output of each block were connected via skip connection. CNN layers with a stride of 2 were applied every 2 or 4 residual blocks. The depth (number of features) of the CNN layers increased by a factor of 2 per 4 residual blocks. For example, a stride described as “2,1,1” in Multimedia Appendix 3 implies that there are 3 CNN layers in that block and the stride of those CNN layers are 2, 1, and 1, respectively. The “Length” and “Depth” columns in Multimedia Appendix 3 are the length and depth of the output of each block. Each feature of the final output of the encoder was average pooled to obtain length=1.

#### Self-attention Phase

To capture the associations among each lead channel, we utilized a multi-head self-attention module that consisted of queries, keys, and values. Each query, key, and value represented a single dense layer that took all output from the encoder (ie, 
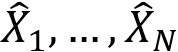
) [24]. We computed the dot products of the query with all keys and applied a softmax function to obtain *N*×*N* attention matrices, where *N* is the number of lead channels. During the training phase of the model, to ensure generalization and applicability for any lead combinations (eg, various 4-, 3-, 2-, and single-lead sets), we randomly masked each lead channel on the attention matrices. Meanwhile, we masked all the lead channels except for the specific targeting leads during the inference phase. For instance, if the target leads were I and V1, we masked all other leads but leads I and V1 during the inference phase. Any lead combinations can be set as target leads. The specific combinations that we tested are specified in Multimedia Appendix 2. After acquiring the attention matrices, we computed the dot products of the values with attention matrices such that the model could reflect the relation between leads. Afterward, these outputs from all the multi-heads were concatenated and linearly projected so that the final output dimension of the multi-head self-attention module became 512 (the same as the original input of the multi-head self-attention module).

We then flattened the output of lead channels before feeding them into the classifier. The classifier had 2 layers of dense layers, which reduced the dimension from 6144 (512 × 12) to 1, followed by a sigmoid layer that calibrated the probability of acute myocardial infarction (ie, 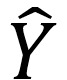) range from 0 to 1. We split the data into training/validation (80%) and independent hold-out test (20%) sets, and then further split the training/validation set into training (85%) and validation (15%) sets. For training, we used the Adam optimizer with a batch size of 32 and a learning rate of 0.001. We also applied weight decay and several data augmentation techniques, including random Gaussian noise, time scaling, and signal masking, to prevent overfitting. To tune the hyperparameters, we utilized validation data sets with extensive experiment settings (ie, 12-, 4-, 3-, 2-, and single-lead settings). We implemented the model using the Pytorch library.

## Results

### Data Set Characteristics

**Figure 3:**
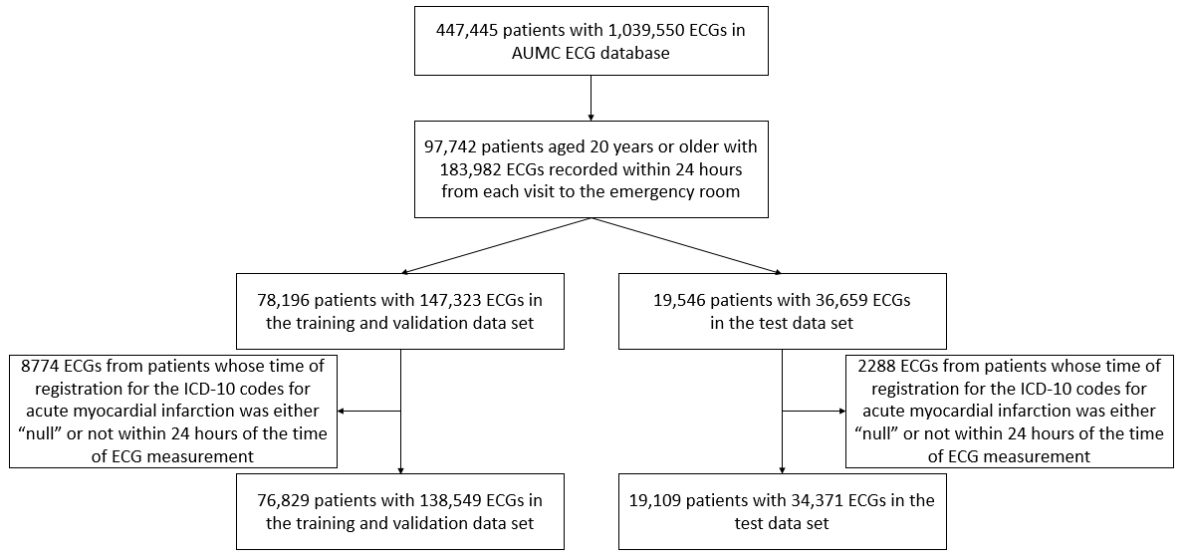
Patient flow diagram. The patients were split into training and validation (80%) and test (20%) data sets. ECG: electrocardiogram, ICD-10: International Classification of Diseases, Tenth Revision.

From the AUMC ECG database, we extracted 97,742 patients aged 20 years or older with 183,982 ECGs recorded within 24 hours from each visit to the emergency room (Figure 3). After applying the exclusion criteria, we included 76,829 patients with 138,549 ECGs in the training and validation data set, and 19,109 patients with 34,371 ECGs in the test data set. The data set characteristics are summarized in Table 1. The proportion of ECGs labeled as acute myocardial infarction was 1.78% for the training and validation data set, and 1.61% for the test data set.

**Table 1:** Data set characteristics.

Characteristics				Training and validation (n=138,549)		Test (n=34,371)
Patients, n				76,829		19,109
Age (years), mean (SD)				59.00 (16.98)		59.00 (16.95)
**Sex**						
	**Male**					
		Electrocardiographs, n	75,552		18,426	
		Patients, n	40,662		10,043	
	**Female**					
		Electrocardiographs, n	64,097		15,945	
		Patients, n	36,170		9066	
Acute myocardial infarction, n				2465		554

### Model Performance

**Figure 4:**
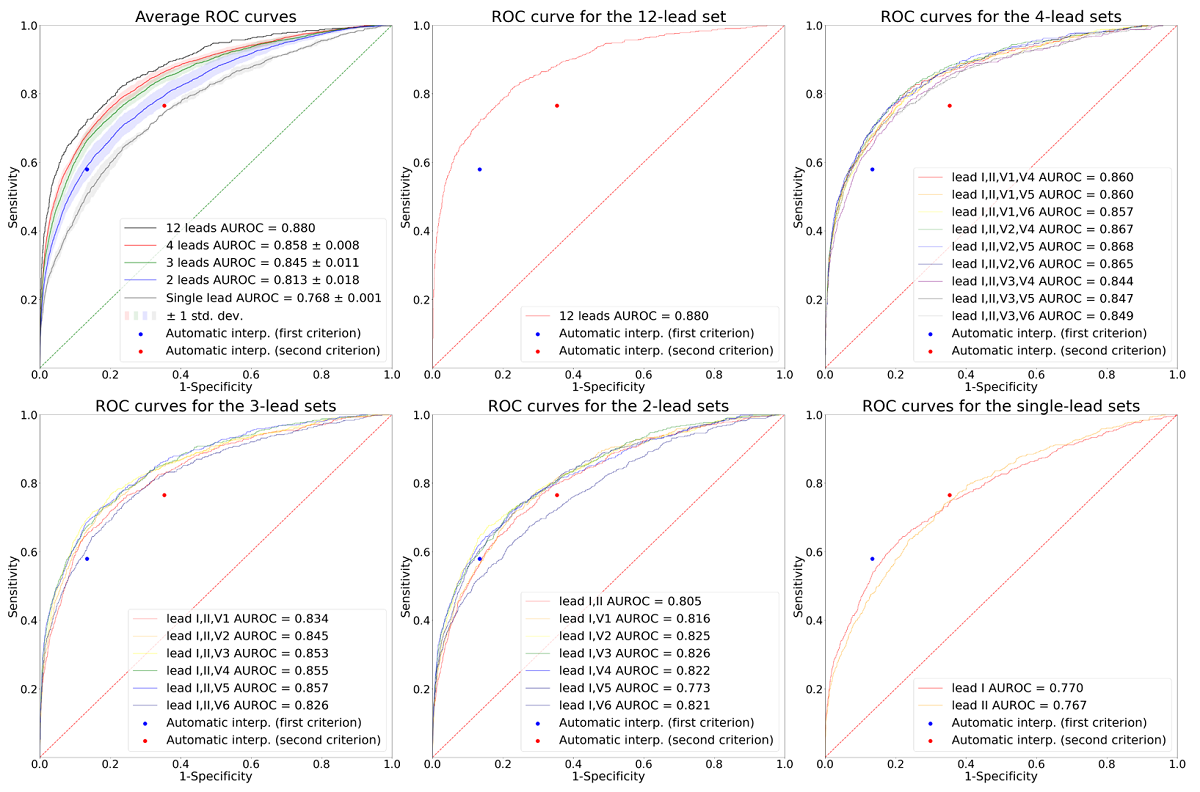
ROC curves for the various target lead sets. The plot on the upper left shows the average ROC curves according to the number of leads. The solid lines depict the average ROC curves, and the shaded areas depict 1 SD of the ROC curves. The rest of the plots show the ROC curves for the 12-, 4-, 3-, 2-, and single-lead sets, respectively. In all plots, the performance of the automatic ECG interpretations is depicted as dots. AUROC: area under the receiver operating characteristic curve; ROC: receiver operating characteristic curve.

**Figure 5:**
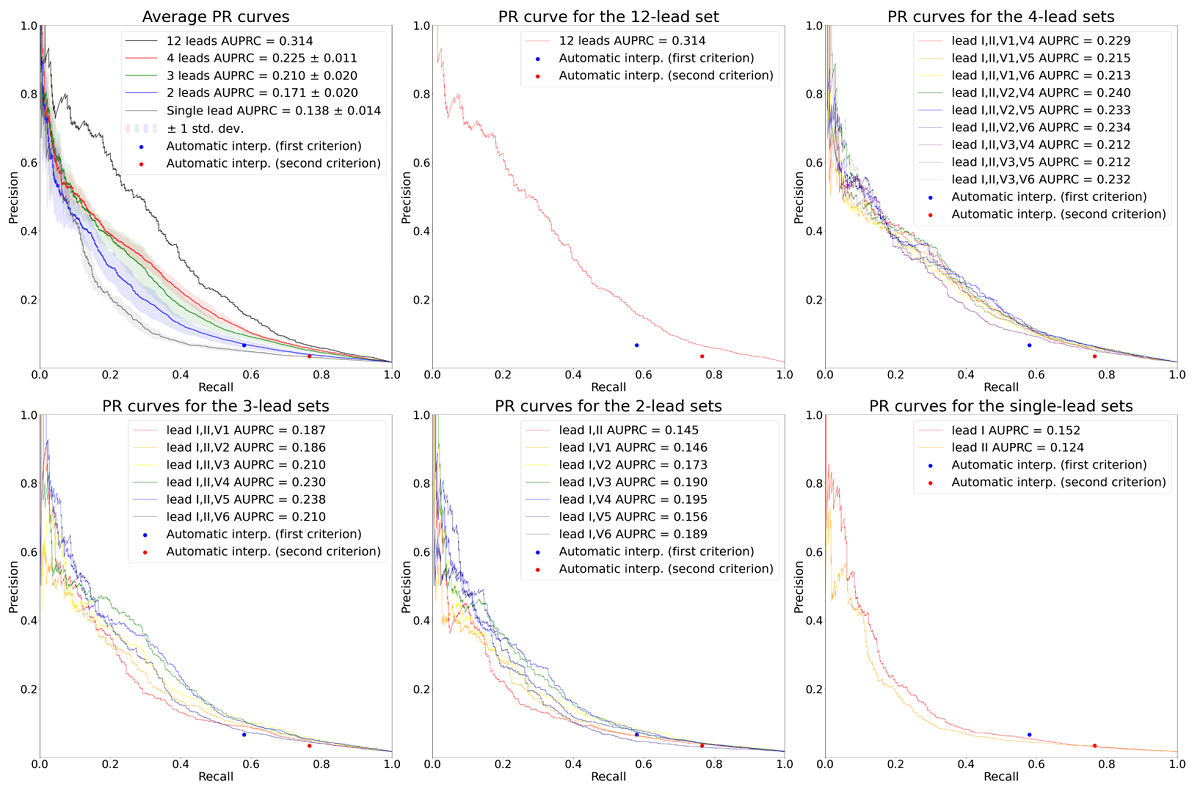
PR curves for the various target lead sets. The plot on the upper left shows the average PR curves according to the number of leads. The solid lines depict the average PR curves, and the shaded areas depict 1 SD of the PR curves. The rest of the plots show the PR curves for the 12-, 4-, 3-, 2-, and single-lead sets, respectively. In all plots, the performance of the automatic ECG interpretations is depicted as dots. AUPRC: area under the precision-recall curve; PR: precision-recall.

Figures 4 and 5 show the receiver operating characteristic (ROC) and the precision-recall (PR) curves for the various target lead sets. The dots indicate the performance of the automatic ECG interpretations provided by the GE ECG analysis program. The sensitivity, specificity, PPV, and NPV of the first labeling criterion of the automatic ECG interpretation were 0.579, 0.866, 0.066, and 0.992, respectively. The corresponding values of the second labeling criterion of the automatic ECG interpretation were 0.765, 0.647, 0.034, and 0.996, respectively. Lead sets with 3 or more leads had a better performance than the automatic interpretations: their corresponding ROC and PR curves consistently lay above the corresponding dots of the automatic ECG interpretations. Similarly, the single-lead sets had worse performance than the automatic ECG interpretations: the corresponding ROC and PR curves lead sets lay below the corresponding dots of the automatic ECG interpretations. For the 2-lead sets, some of the ROC and PR curves lay above and some below the corresponding dots of the automatic ECG interpretations, which implied that not all the 2-lead sets had a better performance than the automatic interpretations.

The average AUROCs for the 12-, 4-, 3-, 2-, and single-lead sets were 0.880, 0.858 (SD 0.008), 0.845 (SD 0.011), 0.813 (SD 0.018), and 0.768 (SD 0.001), respectively. The average AUPRCs for the 12-, 4-, 3-, 2-, and single-lead sets were 0.314, 0.225 (SD 0.011), 0.210 (SD 0.020), 0.171 (SD 0.020), and 0.138 (SD 0.014), respectively. These values indicate that the average AUROC and AUPRC increased as the number of leads increased. All the comparisons of AUROCs between ROC curves having the median AUROC from lead sets with different numbers of leads (“12-lead set” vs “4-lead set [leads I, II, V1, V5]” vs “3-lead set [leads I, II, V3]” vs “2-lead set [leads I, V6]” vs “single-lead set [lead I]”) were statistically significant at a significance level of .05, as revealed through the DeLong test [25]. All the comparisons of AUROCs between ROC curves having the highest AUROC from lead sets with different numbers of leads (“12-lead set” vs “4-lead set [leads I, II, V2, V5]” vs “3-lead set [leads I, II, V5]” vs “2-lead set [leads I, V3]” vs “single-lead set [lead I]”) were also statistically significant at a significance level of .05, as revealed through the DeLong test.

When we set the thresholds of the lead sets to match the specificity of the first labeling criteria of the automatic ECG interpretation (specificity=0.866), the 12-, 4-, and 3-lead sets demonstrated an average gain in sensitivity of 13.9%, 10.2% (SD 1.6%), and 8.5% (SD 2.7%), respectively (Table 2), compared to the automatic ECG interpretation, while maintaining a high NPV above 0.99. The results for the second labeling criteria (specificity=0.647) revealed average gains in sensitivity of 11.9%, 9.8% (SD 1.2%), and 8.1% (SD 1.5%) for the lead sets with 12, 4, and 3 leads, respectively (Table 2), while maintaining a high NPV above 0.99. The sensitivities of the 2-lead sets were, on average but not consistently, slightly higher than those of the automatic ECG interpretations when the thresholds of the 2-lead sets were set to match the specificities of the automatic ECG interpretations. Single-lead sets had lower sensitivities than the automatic ECG interpretations when the specificities were matched.

**Table 2:** Average sensitivity, positive predictive value, and negative predictive value according to the number of leads when the thresholds were set to match the specificity of the first or second labeling criteria of automatic electrocardiogram interpretation.

	Sensitivity			Positive predictive value			Negative predictive value	
	At specificity=0.866 (first labeling criteria)	At specificity=0.647 (second labeling criteria)	At specificity=0.866 (first labeling criteria)		At specificity=0.647 (second labeling criteria)	At specificity=0.866 (first labeling criteria)		At specificity=0.647 (second labeling criteria)
Automatic electrocardiogram interpretation	0.579	0.765	0.066		0.034	0.992		0.996
12-lead set	0.718	0.884	0.081		0.039	0.995		0.997
4-lead sets, mean (SD)	0.681 (0.016)	0.863 (0.012)	0.077 (0.002)		0.039 (0.001)	0.994 (0.000)		0.997 (0.000)
3-lead sets, mean (SD)	0.664 (0.027)	0.846 (0.015)	0.075 (0.003)		0.038 (0.001)	0.994 (0.000)		0.996 (0.000)
2-lead sets, mean (SD)	0.589 (0.038)	0.794 (0.030)	0.067 (0.004)		0.036 (0.001)	0.992 (0.001)		0.995 (0.001)
Single-lead sets, mean (SD)	0.505 (0.029)	0.745 (0.001)	0.058 (0.003)		0.033 (0.000)	0.991 (0.001)		0.994 (0.000)

## Discussion

### Principal Findings

In this study, we developed an AI model for detecting acute myocardial infarction by randomly masking each lead channel during the training phase and testing the model using various target ECG lead sets with the remaining lead channels masked. First, we found that the performances of lead sets with 3 or more leads compared favorably with that of the automatic ECG interpretations provided by the GE ECG analysis program, with a 8.1%-13.9% gain in sensitivity when the threshold was set to match the specificity of the automatic ECG interpretations, and with the ROC and PR curves lying above the corresponding dots of the automatic ECG interpretations. Only some of the 2-lead sets compared favorably with the automatic ECG interpretations. When only a single lead was evaluated, acute myocardial infarction could be underdiagnosed; thus, useful information from other leads could potentially be neglected. Indeed, single-lead sets performed worse than the automatic ECG interpretations.

Multiple-lead ECG is necessary for the accurate and robust detection of cardiac disorders, particularly acute myocardial infarction. Given that multiple-lead ECGs can be obtained by smartwatches only in an asynchronous manner, our results imply that multiple lead-based AI-enabled ECG algorithms can be implemented on these devices. Such implementation could facilitate timely diagnostics to enhance outcomes and reduce mortality among cardiovascular disease populations outside the hospital.

Second, we found that model performance generally increased as the number of leads increased (12-lead set: AUROC 0.880; 4-lead sets: AUROC 0.858, SD 0.008; 3-lead sets: AUROC 0.845, SD 0.011; 2-lead sets: AUROC 0.813, SD 0.018; single-lead sets: AUC 0.768, SD 0.001). With smartwatches, measuring additional leads would only take less than a minute, and the benefit of doing so would greatly outweigh the risk. In an emergency situation, we suggest measuring at least 3 leads (ie, I, II, and V5) and ideally more than 4 leads (ie, I, II, V2, and V5) to minimize the risk of failing to detect acute myocardial infarction occurring in a certain spatial location or direction.

Previous studies on automated diagnosis or classification of multiple-lead ECGs using AI have used synchronous ECG signals as input. The results from these studies are insufficient for the evaluation of the feasibility of multiple lead-based AI-enabled ECG algorithms on smartwatches since only asynchronous ECG signals can be obtained from smartwatches. To the best of our knowledge, our study is the first to utilize asynchronous ECG signals for AI model development. Future studies could aim at developing AI models with asynchronous ECG signals for detecting cardiac disorders other than acute myocardial infarction, such as cardiac arrhythmias or contractile dysfunctions.

Our study has important medical and economic impacts. First, our model can significantly reduce time to diagnosis, and consequently reduce time to reperfusion, which is the elapsed time between the onset of symptoms and reperfusion and is critical to the clinical outcome of the disease [26]. The ECG is commonly the first diagnostic test in the evaluation of myocardial infarction, and it should be acquired as early as practicable [27]. Traditionally, the bulky ECG equipment and the need for a trained physician for diagnosis have required the transfer of patients to hospitals, even in emergency situations. This practice greatly delays time to diagnosis, which would be most ideal if made directly in the field. With our model implemented on smartwatches, reliable preliminary diagnosis can be made even before contact with emergency services, thereby greatly reducing the time from the onset of symptoms to diagnosis. With the preliminary diagnosis already made, patients can be promptly triaged to the most appropriate form of treatment after accounting for geographical factors and available facilities [26]. The final diagnosis should be made by a trained physician after arriving at the appropriate facility, but with the aid of our model, the time required for the entire process can be greatly reduced. The threshold for a positive result from our model can be altered to balance between over- and undertriage. Second, our model has the potential to greatly reduce mortality and the related economic burden due to acute myocardial infarction. Untimely diagnosis or treatment results in increased myocardial damage and mortality. The extent of myocardial salvage is greatest if patients are reperfused in the first 3 hours after onset of symptoms [28]. For every 30-minute delay in coronary reperfusion, the relative 1-year mortality rate increases by 7.5% [29]. Our model can reduce mortality due to acute myocardial infarction by facilitating timely diagnosis and reperfusion. Consequently, the economic cost caused by lost productivity from premature mortality due to acute myocardial infarction, which is estimated to be US $40.5 billion annually in the United States, can also be reduced [30]. Third, since our study indicates the feasibility of multiple lead-based AI-enabled ECG algorithms on smartwatches, it can promote the development of AI models with asynchronous ECG signals for detecting cardiac disorders other than acute myocardial infarction, thus accelerating market growth in this field.

### Strengths and Limitations

Our study has several strengths. First, our model only takes ECG as input and does not require other additional clinical data. This implies that our model is highly applicable in real-world, real-time settings where no medical practitioners are available. Smartwatches are the only requirement for applying our model. Second, our model is theoretically implementable with all smartwatches, which further strengthens our study in terms of real-world applicability. That is, creating a mobile software app that activates the ECG hardware, instructs the wearer on how to measure the leads, preprocesses the measured leads to satisfy the input conditions of our AI model (eg, resampling the ECG to 250 Hz, snipping 2.5 seconds from each lead), and runs our AI model, would be sufficient for real-world implementation. We believe that with the aid of mobile app developers, such an app would not be technically difficult to develop. We leave this as a subject for further study. Third, we did not exclude ECGs on the basis of waveform abnormalities. This implies that our model is applicable regardless of ECG abnormalities, thereby greatly enhancing the generalizability to real-world settings. Fourth, our model was trained, validated, and tested with a very large data set of 172,920 ECGs recorded from 95,938 patients. A large enough data set can reduce overfitting to the training set, thus increasing generalizability to other data sets [31,32]. Fifth, as mentioned in the Methods section, our model is applicable to any lead combinations (eg, various 4-, 3-, 2-, and single-lead sets). This is because we randomly masked each lead channel on the attention matrices during the training phase. Thus, users would be able to choose any lead combination in accordance with their preferences or situation.

However, our study also has some limitations. First, our labeling method might be problematic. The diagnosis of acute myocardial infarction does not ensure that the patient’s initial ECG in the emergency room would show explicit signs of acute myocardial infarction. Thus, some ECGs labeled as acute myocardial infarction in our data set might not explicitly show signs of acute myocardial infarction. Nevertheless, our model showed high performance, with our 12-lead set having an AUROC of 0.880. Second, the 12-lead set is not completely asynchronous. When grouped into 4 subsets with 3 leads in each subset, the ECGs are asynchronous intersubset-wise, while being synchronous intrasubset-wise. Thus, the maximum number of leads that can compose a completely asynchronous lead set in our study was 4. The diagnostic capacity of a model tested with 5 or more completely asynchronous lead sets needs to be evaluated in future studies. Third, our model cannot be deemed as a confirmatory test. The final confirmatory diagnosis should be made by a trained physician after the patient arrives in hospital. However, with the preliminary diagnosis made by our model, patients can be efficiently triaged to get the most appropriate form of treatment after accounting for geographical factors and available facilities, even before contact with emergency services. Finally, our model was not validated with external data sets. In future studies, external validation should be performed to ensure the reliability of our model in new environments.

### Conclusions

In conclusion, this study shows the feasibility of multiple lead-based AI-enabled ECG algorithms on smartwatches for the automated diagnosis of cardiac disorders by developing an AI model for detecting acute myocardial infarction with asynchronous ECG signals. We also showed that measuring at least 3 leads, and ideally more than 4 leads, is necessary for accurate detection. Our results show that single-lead sets lack diagnostic performance. From our results, we look forward to the development of other AI models that detect various cardiac disorders using sequentially measured, asynchronous ECG leads from smartwatches. Such models, along with our model, can facilitate timely diagnostics to enhance outcomes and reduce mortality among various cardiac disease populations outside the hospital.

## Appendix

Multimedia Appendix 1 Standard 12-lead ECG report example.

Multimedia Appendix 2 Tested lead sets.

Multimedia Appendix 3 Architecture of the encoder.

## Abbreviations

AI: artificial intelligence
AUMC: Ajou University Medical Center
AUPRC: area under the precision-recall curve
AUROC: area under the receiver operating characteristic curve
CNN: convolutional neural network
ECG: electrocardiogram
GE: General Electric
NPV: negative predictive value
PPV: positive predictive value
PR: precision-recall
ROC: receiver operating characteristic
