# Supplementary figures and images for: Automated Detection of Acute Myocardial Infarction Using Asynchronous Electrocardiogram Signals—Preview of Implementing Artificial Intelligence With Multichannel Electrocardiographs Obtained From Smartwatches: Retrospective Study
Source: J Med Internet Res. 2021 Sep 10;23(9):e31129. doi: 10.2196/31129 (PMC8463948; doi:10.2196/31129)

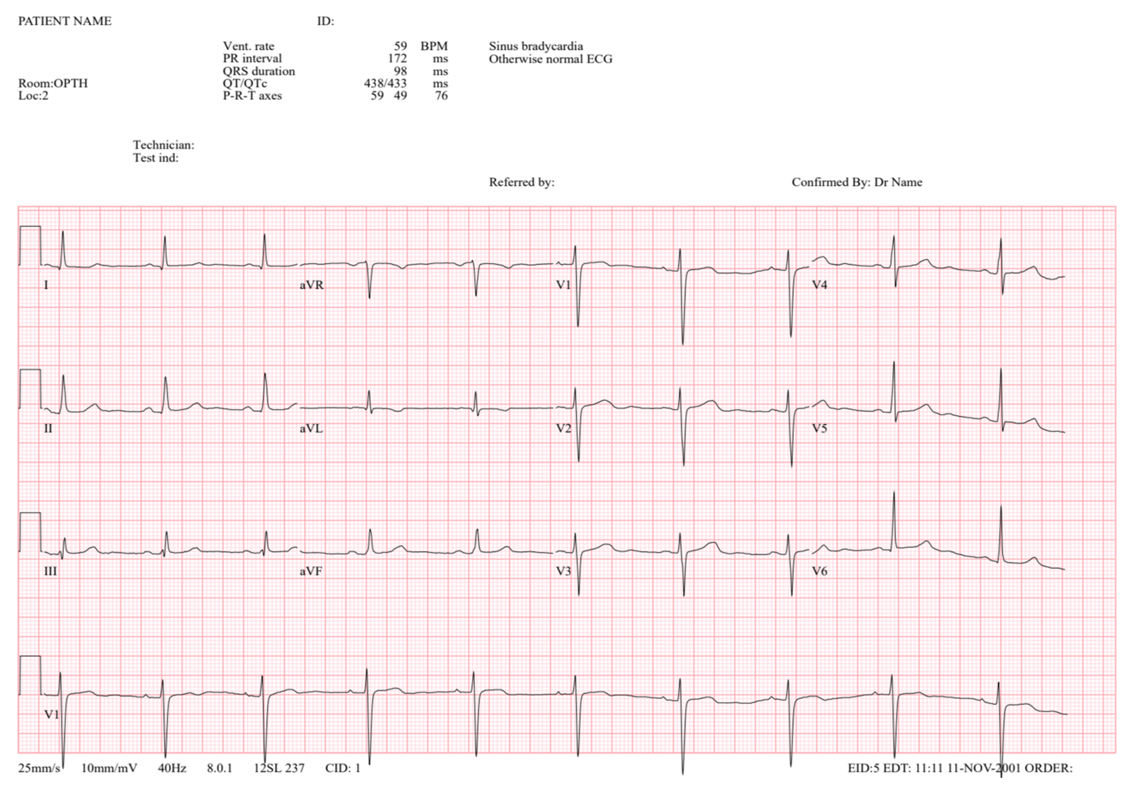

Supplement: Multimedia Appendix 1 [file jmir_v23i9e31129_app1.png]
