# Supplementary material for: Automated Detection of Acute Myocardial Infarction Using Asynchronous Electrocardiogram Signals—Preview of Implementing Artificial Intelligence With Multichannel Electrocardiographs Obtained From Smartwatches: Retrospective Study
Source: J Med Internet Res. 2021 Sep 10;23(9):e31129. doi: 10.2196/31129 (PMC8463948; doi:10.2196/31129)
Supplement: Multimedia Appendix 2 [file jmir_v23i9e31129_app2.docx]

## Multimedia Appendix 2: Tested lead sets.

|  | Leads |
| --- | --- |
| **12-lead set (partially asynchronous)** | (I, II, III, aVR, aVL, aVF, V1, V2, V3, V4, V5, V6) |
| **4-lead sets** | (I, II, V1, V4), (I, II, V1, V5), (I, II, V1, V6), (I, II, V2, V4), (I, II, V2, V5), (I, II, V2, V6), (I, II, V3, V4), (I, II, V3, V5), (I, II, V3, V6) |
| **3-lead sets** | (I, II, V1), (I, II, V2), (I, II, V3), (I, II, V4), (I, II, V5), (I, II, V6) |
| **2-lead sets** | (I, II), (I, V1), (I, V2), (I, V3), (I, V4), (I, V5), (I, V6) |
| **Single-lead sets** | (I), (II) |

The 12-lead set is not completely asynchronous. Each 4-, 3-, and 2-lead set is completely asynchronous.
