# Supplementary material for: Automated Detection of Acute Myocardial Infarction Using Asynchronous Electrocardiogram Signals—Preview of Implementing Artificial Intelligence With Multichannel Electrocardiographs Obtained From Smartwatches: Retrospective Study
Source: J Med Internet Res. 2021 Sep 10;23(9):e31129. doi: 10.2196/31129 (PMC8463948; doi:10.2196/31129)
Supplement: Multimedia Appendix 3 [file jmir_v23i9e31129_app3.docx]

## Multimedia Appendix 3: Architecture of the encoder.

|  | Strides | Length | Depth |
| --- | --- | --- | --- |
| **Input** |  | 625 | 1 |
| **Residual block #1** | [1,1,1] | 625 | 64 |
| **Residual block #2** | [1,1] | 625 | 64 |
| **Residual block #3** | [2,1] | 313 | 64 |
| **Residual block #4** | [1,1] | 313 | 64 |
| **Residual block #5** | [2,1,1] | 157 | 128 |
| **Residual block #6** | [1,1] | 157 | 128 |
| **Residual block #7** | [2,1] | 79 | 128 |
| **Residual block #8** | [1,1] | 79 | 128 |
| **Residual block #9** | [2,1,1] | 40 | 256 |
| **Residual block #10** | [1,1] | 40 | 256 |
| **Residual block #11** | [1,1] | 40 | 256 |
| **Residual block #12** | [1,1] | 40 | 256 |
| **Residual block #13** | [2,1,1] | 20 | 512 |
| **Residual block #14** | [1,1] | 20 | 512 |
| **Residual block #15** | [1,1] | 20 | 512 |
| **Residual block #16** | [1,1] | 20 | 512 |
